# Global Marine LNG Terminals, Tankers & Trade: A High-Resolution AIS-Based Dataset of LNG Trade (2020–2024)

**DOI:** 10.1038/s41597-026-07454-2

**Published:** 2026-05-19

**Authors:** Chuanlong Zhou, Philippe Ciais, Rohith Teja Mittakola, Biqing Zhu, Yang Su, Yidi Xu

**Affiliations:** 1 Laboratoire des Sciences du Climat et de l’Environnement (LSCE), Université Paris-Saclay, CEA-CNRS-UVSQ, Gif-sur-Yvette, France; 2 International Institute for Applied Systems Analysis (IIASA), Laxenburg, Austria; 3 UMR ECOSYS, INRAE AgroParisTech, Université Paris-Saclay, Gif-sur-Yvette, Thiverval-Grignon France

**Keywords:** Physical oceanography, Energy security, Energy supply and demand, Energy policy

## Abstract

We introduce a daily-resolution dataset capturing global maritime liquefied natural gas (LNG) trade flows from 2020 to 2024, derived from Automatic Identification System (AIS) vessel tracking and port call records. The dataset, LNG-T3, integrates LNG tanker fleet specifications, LNG terminal metadata, and AIS-based vessel position and draught reports to map global maritime LNG trade flows. It provides individual LNG tanker export and return voyage events, as well as daily LNG volumes for tanker transport, terminal throughput, and country-to-country trade. The LNG-T3 dataset reveals the major shift in global LNG trade following the 2022 Russian invasion of Ukraine, including a redirection of exports from China to the EU and increased Russian LNG exports to Europe despite the conflict. The United States became the world’s largest LNG exporter, boosting exports to Europe to replace Russian pipeline gas. By offering comprehensive tanker, infrastructure, and trade information, the LNG-T3 dataset enables future analyses of market dynamics, infrastructure bottlenecks, broader geopolitical factors shaping international LNG trade, and climate impacts of the LNG industry.

## Background & Summary

The global maritime trade of liquefied natural gas (LNG) has expanded rapidly in recent years, with total volumes increasing by roughly 30% from 2018 to 2023, rising to about 402 million tonnes in 2023^1,2^. This surge is driven by both rising LNG supply and growing demand. The primary LNG supply expansion came from the United States^3^, which became the world’s largest LNG exporter in 2023 and represents 21% of the global supply^3,4^. On the demand side, the energy demand in Europe has dramatically altered gas markets, as its gas supply shifted from pipeline gas to LNG^5^. Following the 2022 gas crisis triggered by Russia’s invasion of Ukraine^6^, the European Union (EU) boosted its seaborne LNG imports from 80 bcm (billion cubic meters) in 2021 to 130 bcm in 2022—marking a 60% surge as noted by the IEA^7^. At the same time, China, the world’s largest single LNG importer, continued to increase its use of natural gas as a “bridge fuel” to shift away from coal in support of emissions-reduction goals^8,9^, further intensifying the global LNG demand. As a result, the development of new LNG export and import terminals has been fast-tracked worldwide^10,11^. Europe, for example, increased its LNG import capacity by over 30% between 2022 and 2023 by building new terminals^12^. In parallel, the global LNG carrier fleet expanded significantly, adding 41 new ships during 2023 alone^13^.

Despite LNG’s increasingly pivotal role in regional energy security and climate mitigation strategies^5,14^, publicly available data for LNG trade with high spatial-temporal resolution remain limited. Official and industry sources typically publish LNG statistics only as annual or monthly aggregates at the country level^15–18^. Detailed and timely data are mostly sourced from commercial services and databases, making it difficult for researchers and policymakers to perform granular analyses^13,19^. This data gap narrows the scope and depth of potential research, particularly for understanding the short-term fluctuations and region-specific complexities. For example, Zhou *et al*. highlighted the pivotal role of LNG imports in the EU’s response to the gas crisis^5^, however, the lack of high-frequency, source-specific LNG data limits deeper analysis of gas supply source-country diversification, intra-EU LNG redirection, and overall gas system resilience.

Recent research demonstrates that vessel activity tracking with AIS (Automatic Identification System) provides high-temporal-resolution data on both vessel movements and inferred terminal operations. For instance, Yan *et al*. reconstructed LNG port operation activity in China using AIS data and analyzed the optimization potential based on the daily and weekly patterns^8^. Similarly, Chen *et al*. leveraged AIS data to map the global seaborne LNG trade network and pinpoint key terminals within it^20^. Such high-resolution data at the vessel level is essential for analyzing both regional and global LNG trade dynamics, understanding the driving factors such as shifting regional demand, climate variability, and geopolitical events^8,20,21^, and ultimately informing energy policy decisions^9^.

However, previous studies did not release their underlying AIS or aggregated activity data for their analysis^8,21,22^, probably due to their heavy reliance on commercial historical AIS datasets, such as Spire Maritime^23^. This lack of data transparency limits reproducibility and hinders further research. Although researchers can obtain the real-time AIS feeds freely through platforms like AISHub^24^, filtering and interpreting the large-scale raw data requires a well-structured inventory of LNG-specific infrastructure—such as detailed tanker specifications and terminal locations—which is currently lacking. This infrastructure data gap further restricts high-resolution analyses of global LNG trade flows and constrains deeper evaluations of market dynamics, energy security, and climate impacts^25^.

Therefore, we developed the LNG-T3 (Liquefied Natural Gas from Tanker, Terminal, and Trade) dataset, the first open-access dataset to provide global LNG infrastructure and trade flows at a daily resolution from 2020 to 2024. We first collected and integrated data from multiple open sources to construct a comprehensive inventory of the global LNG tanker fleet and metadata for LNG terminals. This inventory was then used to extract LNG tanker operations from real-time AIS vessel position and draught reports and historical port call records (prior to 2023). These operations are compiled into a high-resolution dataset of 17,592 individual tanker voyage events. By aggregating the vessel-level activities, including loading, unloading, and moving, the LNG-T3 dataset offers daily estimates of LNG volumes transported by individual tankers, processed at specific terminals, and traded between countries. The LNG-T3 dataset captures both the physical infrastructure and high-resolution trade dynamics, providing a robust foundation for regional-to-global analyses related to LNG market dynamics, energy security, and climate impacts.

## Method

**Fig. 1:**
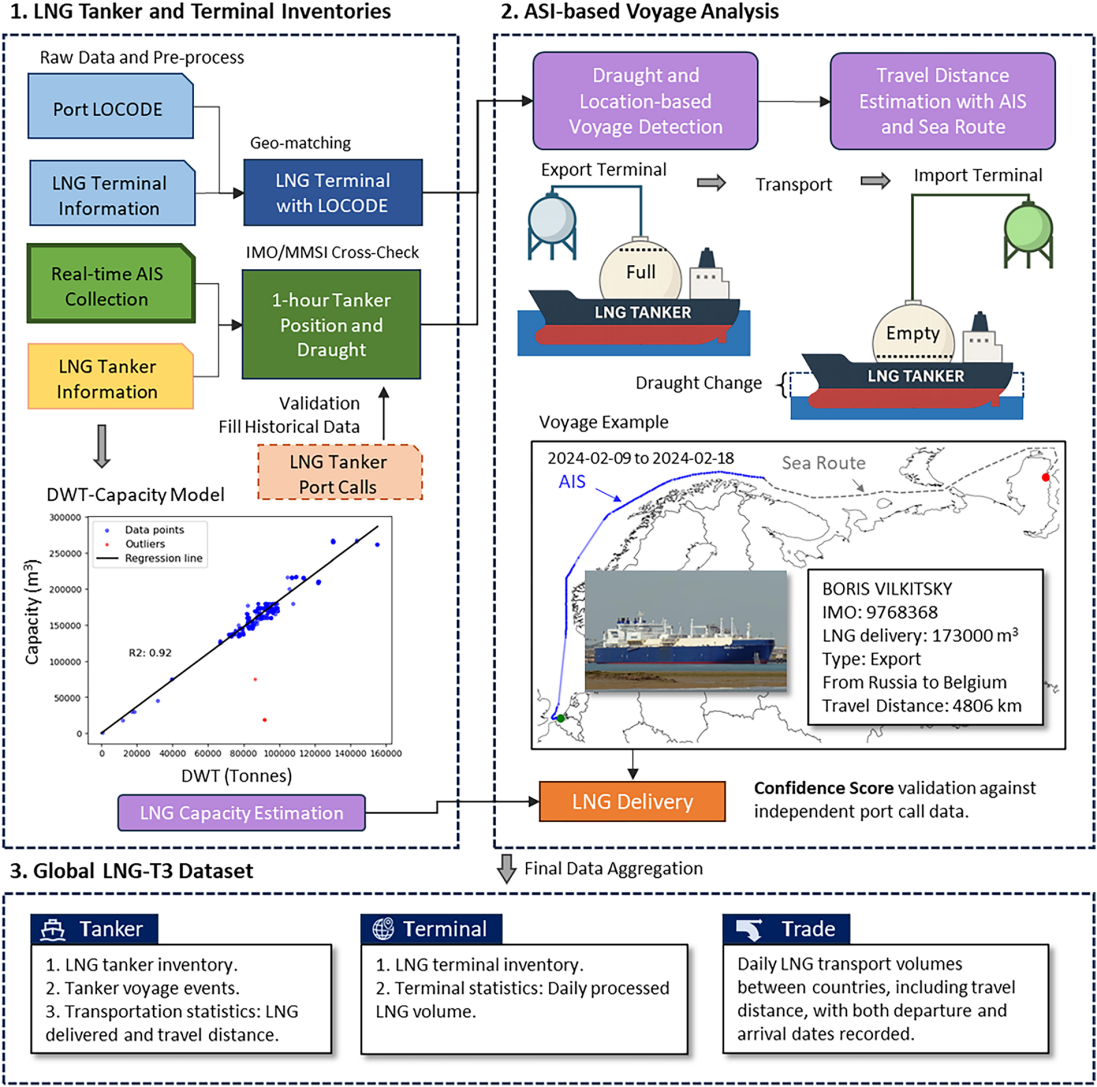
Data acquisition and processing framework.

We constructed the LNG-T3 dataset by systematically collecting, integrating, and processing data from multiple open-access sources, including LNG tanker fleet specifications, LNG terminal metadata, and real-time vessel AIS report data. Figure 1 illustrates the data acquisition and processing framework, which consists of three major components: (1) the compilation of LNG tanker and terminal inventories, (2) AIS-based voyage detection and LNG volume estimation, and (3) the aggregation of the final dataset. The key data sources and software toolkits used are summarized in Table 1. Below, we describe each step of the methodology in detail. Our goal was to accurately map global LNG flows by tracking individual tankers’ activities – loading, unloading, and transiting – and then aggregating those events into meaningful trade volume data at the LNG tanker, terminal, and country trade levels, as the LNG-T3 dataset. Notably, this framework is fundamentally based on open AIS data, which can be adapted to analyze the transportation events of other vessel categories beyond the LNG fleet.

**Table 1:** Summary of open-access data, commercial products, and toolkits used in constructing the LNG-T3 dataset.

Name	Type	Usage in Study	Source/Access
AISHub	Open-access	Real-time vessel geolocation and draught information (2023 onward)	https://www.aishub.net
VesselFinder Port Call*	Commercial**	Historical vessel entry/exit events at ports (2020–2022)	https://www.vesselfinder.com
Nakilat Fleet	Open-access	LNG tanker metadata	https://www.nakilat.com/fleet-list-q-flex/
Helderline	Open-access	LNG tanker metadata	https://www.helderline.com/tanker/
ShipVault	Open-access	LNG tanker metadata	https://www.shipvault.com
VesselFinder	Open-access	LNG tanker metadata	https://www.vesselfinder.com
Global Energy Monitor	Open-access	LNG terminal metadata	https://globalenergymonitor.org
UN/LOCODE	Open-access	Linking LNG terminals with commercial ports	https://unece.org/trade/cefact/unlocode-code-list-country-and-territory
SeaRoute (Eurostat)	Open-access	Estimating missing vessel trajectories	https://github.com/eurostat/searoute

*The LNG-T3 dataset does not contain or redistribute any raw port call records from VesselFinder. Only aggregated, derived voyage-level and trade-level statistics are included in the public release.

^**^For the 2020–2022 period, port call records were utilized to reconstruct historical voyages as this period precedes the active AIS data collection initiated for this study. We include this period to enhance the dataset’s value for analysis of global trade shifts surrounding the 2022 energy crisis. It is important to emphasize that the core methodological framework remains fundamentally based on open-access data, and data from 2023-01 to 2024-12 are generated with AIS data only.

### LNG tanker inventory

We first developed a global LNG tanker fleet inventory to filter AIS records from large raw records. We integrated and cross-validated information from multiple platforms that provide LNG tanker details, including VesselFinder^26^, Nakilat^27^, Helderline^28^, and ShipVault^29^. We merged data from these sources using the International Maritime Organization (IMO) number, which remains constant throughout the vessel’s operational life. We also cross-check the Maritime Mobile Service Identity (MMSI) number and other vessel metadata to further validate data quality. Note that the MMSI number commonly used in AIS reports is not a reliable identifier for long-term tracking, as it may change over time due to flag reassignments or communication system upgrades. We manually review and correct the vessel’s data once the cross-validation fails. To ensure the completeness and reusability of the tanker inventory, we provide the tanker list until March 2026, although the trade data is updated until the end of 2024.

The key attribute of the tanker inventory is the vessel’s transport capacity, which directly determines how much LNG (in volume or mass) a ship can carry per voyage. However, this capacity is not consistently reported in freely available data sources. Instead, the deadweight tonnage (DWT), a standard measure of the total weight a vessel can safely carry, is more commonly available across public maritime platforms^26^. Given the strong correlation between LNG capacity and DWT, we developed a linear regression model (R² = 0.92) to estimate missing capacity values using DWT as the predictor (Fig. 1). Obvious outliers—tankers with unrealistically low LNG capacity relative to their DWT—were corrected using this model to ensure data consistency (highlighted in red). In total, 18 estimations and corrections were applied out of 406 records.

After this step, our final LNG tanker inventory provides, for each vessel, the following attributes (see Tables 2, 3): IMO number, current MMSI, vessel name, year built, vessel class, flag state, LNG transport capacity (m³, cubic meters), whether an active tanker, and an indicator flag if the capacity was estimated from DWT rather than directly reported. We also classified tankers into five capacity-based categories: Q-Max (>250,000 m³), Q-Flex (>200,000 m³), large conventional carriers (>150,000 m³), standard conventional carriers (>125,000 m³), and small LNG carriers (<125,000 m³)^27^. Here, an active tanker is defined as a vessel with at least one record presented in either the AIS dataset or the port call dataset during the study period from 2020 to 2024 (see **Vessel AIS Data** and **Vessel Port Call Data** below). This comprehensive LNG tanker inventory provides essential input for AIS-based vessel filtering, LNG transport capacity estimation, and detailed tanker-level analyses in both this study and future research.

**Table 2:** Key Attributes of LNG Tankers Records in the LNG-T3 Dataset.

Dataset	Attribute	Description
Tankers	IMO	International Maritime Organization number
	MMSI	Maritime Mobile Service Identity number
	ship_name	Name of the LNG tanker
	built_year	The year the vessel was built
	tanker_class	Classification of tankers by size/type
	capacity_cbm*	LNG carrying capacity in cubic meters (m³)
	registered_country	The country where the tanker is registered
	is_capacity_estimated*	Flag indicating if capacity is estimated (1 = Yes)
	is_active_tanker	Flag indicating if the tanker has AIS or port call record during the study period
	total_LNG**	Total LNG volume transported by the vessel (m³)
	delivery_counts	The number of LNG deliveries made by the tanker
	total_distance**	Total vessel travel distance (in kilometers)
	first_delivery_record, last_delivery_record	Date of the earliest and latest recorded LNG delivery
Tanker Voyages	start_date, end_date	Voyage date of departure and arrival
	voyage	Voyage type, export or return
	from_terminal, *to_terminal*	LNG terminal of departure and arrival
	amount_cbm	Delivered LNG cargo volume (m³)
	from_country, to_country	Country of departure and arrival
	confidence_score	Confidence level of detected voyage
	voyage_distance	Estimated voyage distance from origin to destination (in kilometers)

*The fields capacity_cbm and is_capacity_estimated are derived from a DWT-to-capacity regression model (r^2^ = 0.75, see Methods). The model was applied exclusively in cases where official capacity data were not available, and the flag is_capacity_estimated will be set 1.

**“Total” values in the LNG-T3 dataset (e.g., total_LNG, delivery_counts, total_distance, total_processed_cbm) represent aggregated values over the period from 2020-01 to 2024-12, based on available and validated data records.

**Table 3:** Key Attributes of LNG Terminals and Trade Records in the LNG-T3 Dataset.

Terminals	name	Name of the LNG terminal
	UN_LOCODE	UN/LOCODE code of the nearest port to the terminal
	port_distance_km	The distance between the terminal and the port
	status	Operational status (e.g., proposed, idle, operating)
	unit_count	Number of processing units/trains at the terminal
	capacity_mtpa	LNG processing capacity in million tonnes per annum
	terminal_type	Import or export classification
	start_year	Planned or actual start year of terminal operations
	region	Geographical region where the terminal is located
	areas	Country or locality of the terminal
	lat, lon	Geocoordinate of the terminal
	total_processed_cbm*	Total LNG volume processed at the (m³)
	first_record, last_record	Earliest and latest record of terminal activity
Terminal daily	name	LNG terminal name
	date	Date of the activity
	processed_cbm	Total LNG volume processed at the (m³)
Trade	date	Date of LNG trade activity
	type	Type of activity (e.g., arrival or departure)
	from_country	Country of departure
	to_country	Country of arrival
	amount_cbm	Traded LNG cargo volume (m³)
	voyage_distance	Estimated voyage distance (in kilometers)
	confidence_score	Confidence level of the estimated trade event

*“Total” values in the LNG-T3 dataset (e.g., total_LNG, delivery_counts, total_distance, total_processed_cbm) represent aggregated values over the period from 2020-01 to 2024-12, based on available and validated data records.

### Vessel AIS data

**Fig. 2:**
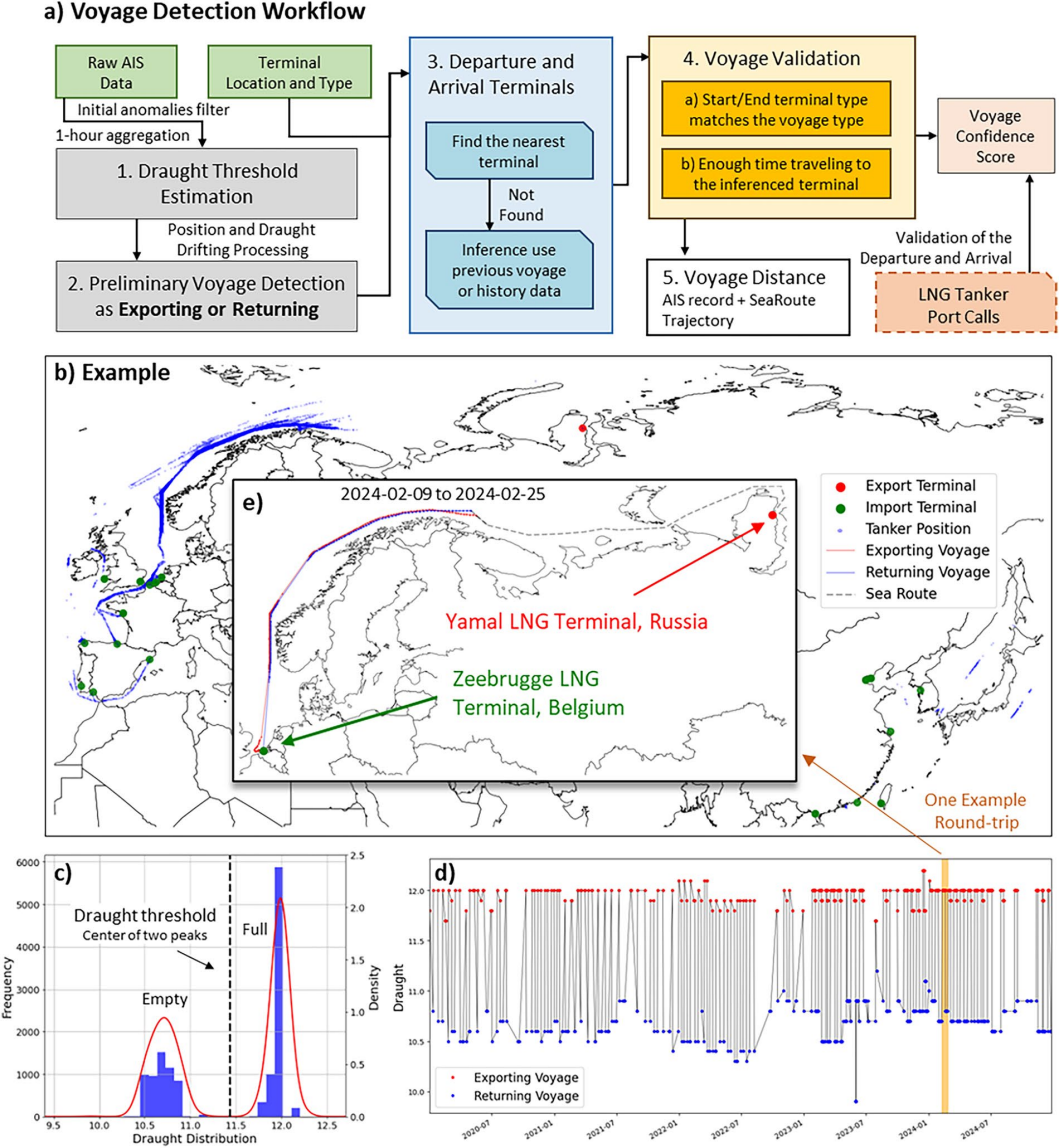
Voyage detection workflow (**a**) and a detailed example for: (**b**) raw AIS data, (**c**) draught threshold analysis, (**d**) detected voyage, and (**e**) one valid voyage example with travel route.

We collected real-time AIS records through the free API provided by AISHub (https://www.aishub.net/api), which offers global vessel AIS reports containing timestamps, geolocation (latitude and longitude), draught, and other voyage-relevant information. We began continuous AIS data collection in early 2023, motivated by the surge in interest in tracking LNG shipments after the 2022 Ukraine-related gas crisis elevated LNG’s strategic importance^6^. Raw AIS records, which contains all vessels, were filtered to include only LNG tankers by matching with the IMO numbers listed in our LNG tanker inventory. The filtered records were harmonized and aggregated at 1-hour intervals (example see Figs. 1, 2e).

We aggregated raw AIS records into a 1-hour temporal resolution to balance computational efficiency with the sensitivity required for event detection. The primary objective of LNG-T3 is to identify discrete trade events (loading and unloading) rather than to provide high-fidelity vessel trajectory tracking. A 1-hour resolution is sufficient to capture LNG cargo operations, which typically span 12 to 36 hours. Within each 1-hour window, we retain the median latitude and longitude to mitigate the impact of spatial drifting and the mode (most frequent) draught value to eliminate transient reporting errors.

### LNG Terminal inventory

We then developed a global LNG terminal inventory to accurately link AIS records to specific trade voyages. We collected the LNG terminal data from the Global Energy Monitor^30^, which provides individual terminal-level webpages containing detailed metadata. We collected and harmonized relevant information from each terminal’s webpage to construct a structured inventory with the following attributes: terminal name, operational status, number of processing units, annual LNG handling capacity (in million tonnes per year, mtpa), terminal type (import/export), start year, geographic region, country, and terminal coordinates (latitude and longitude), as detailed in Table 3.

### Voyage detection

We developed a detection approach that is executed entirely on the raw AIS trajectory and terminal coordinates. Understanding when and where a tanker loads and unloads its cargo is key to constructing the LNG trade flows. We use draught (or draft), the vertical distance between the waterline and the bottom of the ship, as the key parameter to split the load/unload events from continuous AIS records. The underlying assumption is that LNG tankers exhibit significant draught changes during cargo operations: a tanker’s draught increases when it loads LNG and decreases after it unloads.

In summary, we developed a voyage detection framework that tracks draught changes over time with LNG terminal locations (illustrated in Fig. 2a): (1) vessel-specific draught thresholds estimation, (2) preliminary voyage splits with draught, (3) identification of departure and arrival terminals, (4) voyage validation, and (5) voyage route estimation.

We define two types of voyages for the voyage detection framework: exporting voyages, where a vessel departs from an LNG export terminal in a fully loaded state and arrives at an import terminal, and returning voyages, where a vessel departs from an import terminal in an unloaded state and arrives at an export terminal.

To ensure the reliability of this detection framework, we implemented sequential consistency constraints to filter out physically improbable anomalies. Specifically, we prohibit draught “jumps” (e.g., rapid transitions between laden and ballast states) within a 12-hour window, as actual LNG terminal operations require significantly longer durations. Any such transient fluctuations are treated as manual reporting errors. Similarly, for spatial data, we applied a trajectory smoothing logic that identifies and removes “spikes”—records showing temporary spatial drifting that deviates from the vessel’s continuous path. These rules prevent the misclassification of noise as discrete loading or unloading events.

One application of this framework to a representative LNG tanker (*BORIS VILKITSKY*) is illustrated in Fig. 2b–d. The vessel’s draught distribution (Fig. 2c) shows a clear separation between fully loaded and unloaded status. The corresponding draught timeline in Fig. 2d captures the loading–unloading cycle, indicating multiple exporting and returning voyages. An example round-trip voyage (Fig. 2e) begins at the Yamal LNG Terminal in Russia, where the vessel departs at full draught, and proceeds to the Zeebrugge LNG Terminal in Belgium, then returns at reduced draught. The following sections describe each of the processing steps in detail.

### Vessel draught threshold

LNG tankers typically exhibit significant changes in draught when transitioning between fully loaded and unloaded conditions (as shown in the schematic illustration in Fig. 1). However, there is no universal draught threshold that separates loaded from unloaded states, as the observed draught values can vary significantly depending on vessel design, ballast operations, sea conditions, and cargo volumes.

To address this, we adopted a data-driven approach to define vessel-specific draught thresholds. For each tanker, the threshold (*d*_*th*_) was computed as the average of the two dominant peaks in its historical draught distribution, which correspond to the vessel’s typical fully loaded and unloaded states:

1 $${d}_{{th}}=\frac{1}{1}\left({d}_{{empty}}+{d}_{{full}}\right)$$

where *d*_*empty*_ and *d*_*full*_ represent the peak values associated with the most frequently observed loaded and unloaded conditions, respectively. To determine vessel-specific thresholds, we applied Kernel Density Estimation (KDE) to historical draught records using a Gaussian kernel with bandwidth determined by Scott’s Rule. This approach effectively resolves the bimodal distribution characteristic of LNG tankers (representing ballast and laden states) while smoothing out noise from manual reporting. The operational threshold was defined as the midpoint between the two highest density peaks (see example in Fig. 2c). This approach proved robust across the LNG tanker fleet, with the resulting thresholds showing a strong correlation with tanker capacity (R² = 0.73; Fig. 4c).

### Preliminary voyage splits

Using the vessel-specific draught thresholds derived above, we classified all AIS records into binary loading states: fully loaded or unloaded. Based on these classifications, preliminary voyages were identified by tracking sequences of loading-state transitions over time (see example in Fig. 2d). Consecutive records with the same loading status were aggregated into a single voyage segment, unless clear anomalies were found, such as abrupt draught shifts or inconsistent geolocations.

### Departure and arrival terminals

For each voyage segment, the nearest LNG terminals within a certain distance were assigned to the starting and ending records. However, those terminals may be incomplete (no terminal can be found) due to missing data or ambiguous trajectories. To address this, we applied an inference process that integrates spatial proximity, temporal continuity, and vessel-specific historical behavior to reconstruct incomplete voyage information (as shown in Fig. 2a). In brief, missing origin or destination terminals were filled using the terminals from the preceding or following voyages when the time interval was short (less than five days). Otherwise, the most likely terminal was selected based on historical frequency and spatial proximity, considering voyage type (i.e., export or return). For example, if the departure terminal was missing for a separate export voyage, the nearest LNG export terminal to the reported starting location was assigned.

Terminals visited without corresponding draught changes were ignored, under the assumption that no LNG loading or unloading occurred. This design ensures that each identified voyage corresponds to actual LNG delivery or return activity, consistent with our objective of estimating country-to-country LNG trade volumes.

### Vessel port call data

Commercial port call records provide discrete vessel entry and exit events at port zones along with draught information, which can be utilized to directly derive tanker voyages and associated loading or unloading events. Consequently, we incorporated commercial port call records obtained from VesselFinder^26^ (covering 2020-01 to 2024-05) to serve as an independent validation benchmark for our AIS-based algorithm (see **Voyage Validation**). Furthermore, these port call data were used to extend the dataset’s temporal coverage to the period prior to the 2022 energy crisis, which significantly enhances the dataset’s value by enabling a robust comparative analysis of trade dynamics before and after the Russia-Ukraine conflict.

Since port call records utilize UN/LOCODE or port names rather than precise coordinates, we performed geospatial matching between each LNG terminal in our inventory and its corresponding port in the UN/LOCODE database^31^, as many LNG terminals are situated within larger port complexes or share a harbor. These port code mappings for each LNG terminal were subsequently integrated into our terminal inventory. This mapping ensures that both the AIS-driven approach and the port call-based reconstruction share a consistent reference terminal baseline for both validation and temporal extension purposes.

### Voyage validation

The detected voyages with origin and destination terminals were validated by considering both voyage type and route feasibility (Fig. 2a). First, we confirmed that each voyage’s assigned departure and arrival terminals matched its classified voyage type: exporting voyages must originate from export terminals and terminate at import terminals, and vice versa for returning voyages. Second, we checked that the vessel’s travel time and trajectory were consistent with the assigned terminal, ensuring that observed draught changes occurred within a reasonable distance of the LNG terminal. These validation steps were essential for excluding false draught changes resulting from ballasting or other operational factors rather than LNG transfer. Only voyages that satisfied both terminal-type consistency and terminal-location plausibility criteria were retained for the final LNG-T3 dataset. This stage of validation relies exclusively on AIS data to verify the inherent logical consistency of the identified voyages.

We further performed an independent validation using commercial port call data as a ground-truth reference to verify whether valid port call records can be found for both the start (loading) and end (unloading) events of each detected voyage. Therefore, an ideal pattern of LNG delivery is characterized by starts and ends with port calls near an export/import terminal, corresponding AIS-detected loading/unloading events, and a continuous AIS trajectory between terminals. This verification framework ensures that the AIS-based detection results are rigorously cross-referenced against independent commercial records.

Each identified voyage was assigned a confidence score (ranging from 5 to 1, representing high to low confidence) based on the combined completeness of AIS trajectory and port call data. A score of 5 is assigned when both loading and unloading events are validated by port call records and the associated AIS trajectory is complete. A score of 4 is given when both events are validated by port call data, but the AIS trajectory is incomplete or sparse. A score of 3 is assigned when only one event is validated by port call records while the AIS trajectory remains continuous. A score of 2 is given when only one event is validated by port call data and the AIS trajectory is also incomplete. Finally, a score of 1 is assigned when none of the events can be validated by port call records, representing voyages inferred solely from AIS status changes and terminal proximity. These average confidence scores were subsequently utilized to label the overall reliability of country-to-country trade flows.

It should be noted that these confidence scores are primarily meaningful for the period between 2023-01 and 2024-05. For the records prior to 2023, which rely predominantly on port call data for historical reconstruction, the confidence score is consistently assigned as 4. Conversely, for the period following 2024-05, where the dataset is derived exclusively from AIS data without additional commercial port call validation, the score is consistently 1. In instances where the dataset is generated purely based on AIS, voyages are verified solely against terminal-type consistency and terminal-location plausibility criteria to ensure their inherent logical integrity.

### Voyage distance

We also provide vessel travel distance for each valid voyage to support interdisciplinary research such as shipping greenhouse gas (GHG) emissions and maritime economics. To estimate travel distance from the AIS and port call data, the key challenge comes from the data gaps or inconsistencies (see Fig. 2b,e). AIS coverage may be incomplete in certain regions (for instance, in some coastal areas or where vessels turn off AIS transmissions), and port call records don’t provide detailed routes between ports. To address these limitations, we conducted a two-step gap-filling strategy. First, missing AIS segments were interpolated with historical AIS records from the same vessel, using a maximum interpolation radius of 100 km. Second, remaining gaps were reconstructed with the SeaRoute toolkit^32^ to generate the most plausible maritime paths between inferred terminals (illustrated as dashed lines in Fig. 2e).

The *SeaRoute* toolkit developed by Eurostat^32^, which computes the shortest feasible maritime paths between geo-locations based on a predefined global shipping network derived from widely used datasets on international shipping lanes^33,34^. It should be noted that SeaRoute was applied only for validating voyage detected with AIS draught transitions as mentioned above. Therefore, route reconstruction does not influence the identification of loading or unloading events, nor the estimation of LNG trade volumes. Potential uncertainties introduced by route reconstruction only affect voyage distance estimates.

### Final aggregation of the LNG-T3 dataset

The validated vessel voyages were reported in as individual events, and then aggregated into daily country-to-country LNG trade flows by linking the departure and arrival countries of each voyage. We assumed that all LNG tankers departed fully loaded at their rated capacity for each exporting voyage. Each trade flow record includes the date, type (departure or arrival), origin and destination countries, estimated LNG volume (m³), voyage distance (km), and a confidence score described above. In this trade dataset, all voyages departing from or arriving in a country on the same date were aggregated to report total LNG volume, total travel distance, and a weighted confidence score derived from the reliability of the detected voyages. We report arrivals and departures for each country separately because they capture different dimensions of LNG dynamics: departures reflect the supply-side capacity and export strategies of producing countries, while arrivals indicate the demand-side security and dependency of importing countries. This distinction also accounts for temporal differences between shipment departures and arrivals, ensuring that both supply activity and delivery timing can be analyzed consistently.

We also derived daily terminal-level throughput and annual tanker-level deliveries, quantifying the LNG volumes handled by individual facilities and ships. The terminal- and tanker-level data were integrated with the corresponding inventories. Therefore, the LNG-T3 dataset offers a multi-level representation of global LNG trade that links voyages, vessels, terminals, and countries. By capturing both spatial dimensions (terminal and country) and operational dimensions (vessel), it provides a unique resource for analyzing LNG dynamics across scales—from fine-grained assessments of individual voyages and terminal operations to system-wide evaluations of global trade patterns, market shifts, and energy security.

## Data Records

The LNG-T3 dataset can be downloaded from (10.5281/zenodo.17273526). All data files are provided in CSV format using UTF-8 encoding and comma separators. Missing values, where present, are denoted by blank cells. The dataset spans January 2020 through December 2024 and consists of four primary files:

- *LNG_tanker.csv*, which provides the global LNG tanker inventory covering all LNG carrier fleet (861 records up to 2026-03, and 406 tankers have active records during 2020–2024).
- *LNG_tanker_voyage.csv*, which provides the individual LNG tanker export/return voyage events from 2020–2024 (17,592 records).
- *LNG_terminal.csv*, which lists LNG export and import terminals worldwide (545 records).
- *LNG_terminal_daily.csv*, which contains daily terminal-level activity derived from validated voyages (16,115 records).
- *LNG_trade_daily.csv*, which reports daily country-to-country trade flows by linking departure and arrival countries of validated voyages (16,691 records).

The detailed definitions of all variables in these files are discussed in the Methods section, summarized in Tables 2, 3, and fully documented in the dataset repository page Table 4.

**Table 4:** Summary of datasets used for validation for the LNG-T3 dataset.

Data Source	Type	Usage in Study	Source/Access
VesselFinder Port Call	Commercial	Voyage validation from 2023-01 to 2024-05	https://www.vesselfinder.com
OGIM	Open-access	LNG terminal validation	^35^
Gas Infrastructure Europe (GIE)	Open-access	LNG terminal validation	https://www.gie.eu/transparency/databases/lng-database/
International Group of Liquefied Natural Gas Importers (GIIGNL)	Might be limited	LNG trade validation	Data extracted, with files uploaded along with the dataset
U.S. Energy Information Administration	Open-access	LNG trade validation in U.S.	https://www.eia.gov/dnav/ng/hist/ngm_epg0_eve_nus-z00_mmcfm.htm
EuroStat	Open-access	LNG trade validation in EU countries	https://ec.europa.eu/eurostat/databrowser/view/nrg_cb_gasm/default/table?lang=en

In addition, for validation purposes, we provide a supplementary CSV file containing annual LNG trade volumes manually extracted and digitized from GIIGNL reports (*GIIGNL_annual.csv*), as the official documents might be restricted for open access^13,15^.

## Technical Validation

### Compare with reference data

To ensure the accuracy and completeness of LNG-T3, we validated the dataset against multiple independent references at the terminal, country, and regional levels, as summarized in Table 4.

#### Tankers

We cross-validated our tanker inventory against the 2023 GIIGNL Annual Report^15^. Our dataset includes 602 transport tankers with a total capacity of 97 million m³, closely matching the 615 vessels reported by GIIGNL until the end of 2022. There is a 10.5% difference in total capacity, which is attributed to the fact that GIIGNL report includes 49 Floating Storage Regasification Unit (FSRU) in its fleet count; these units are primarily dedicated to stationary storage/regasification and were excluded from our voyage-tracking analysis to avoid overestimating active maritime transport capacity.

#### Terminals

**Fig. 3:**
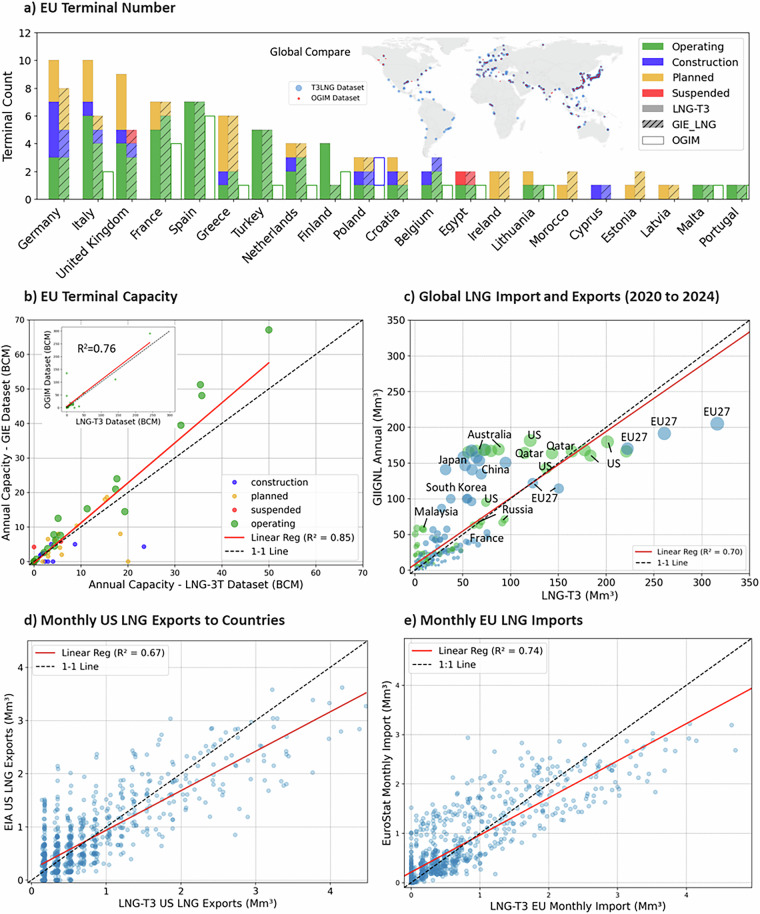
Data Validation for (**a**) EU LNG terminal number, (**b**) EU LNG terminal capacity, (**c**) global LNG trades for 2022 and 2023, (**d**) monthly US LNG exports, and (**e**) monthly EU LNG imports. Data on LNG capacity under construction are shown until December 2024. The inserted map show the spatial comparison between LNG-T3 and OGIM dataset.

We compared our global LNG terminal inventory with the Oil and Gas Infrastructure Mapping (OGIM) database^35^, which documents 338 LNG facilities worldwide for their types, capacities, and locations. The LNG-T3 inventory includes a larger number of terminals overall than OGIM, as we collected more recent data as the LNG industry expanded rapidly. However, for existing facilities, capacity and status classifications show strong agreement with OGIM (R² = 0.76; Fig. 3b, **inserted**). To further validate regional coverage, we compared the European subset of LNG-T3 terminals with the Gas Infrastructure Europe (GIE)^17^. The comparison demonstrates excellent consistency in both terminal counts and capacities (R² = 0.85; Fig. 3a–b). Together, these validations indicate that LNG-T3 provides a comprehensive and reliable representation of global LNG terminal infrastructure, although rapid terminal expansion after 2022 may introduce inconsistencies.

#### Trade between countries

We aggregated LNG-T3 trade flows into annual totals for 2022 and 2023 across major countries and regions and compared them with global statistics published by the International Group of Liquefied Natural Gas Importers (GIIGNL)^13,15^. LNG-T3 values show strong agreement with GIIGNL annual statistics, with country–year pairs exhibiting a high correlation (R^2^ = 0.70, Fig. 3c). At the aggregate level, total export volumes differ from GIIGNL values by 5.1% on average per year, and total import volumes differ by 3.1% per year, primarily due to the voyage validation criteria and incomplete AIS coverage collected in this study. Specifically, for the year 2023, LNG-T3 identified a total global trade volume of 775.0 million m^3^. Using a conversion factor of 0.47 tonnes per m^3^, this equates to approximately 364.2 million tonnes, which captures 91% of the 402 million tonnes reported by GIIGNL^1,2^. Note that these official documents might become restricted for open accessible, underscoring the importance of LNG-T3 in providing open and transparent data for the research and policy community.

At the monthly scale, LNG-T3 flows were further compared with official statistics for key markets. For the United States, LNG-T3 monthly export volumes were well correlated with U.S. EIA statistics (R^2^ = 0.67, Fig. 3d), and the annual totals differed by **1.5%**^16^. For the European Union, LNG-T3 monthly import volumes were strongly consistent with Eurostat data (R^2^ = 0.74, Fig. 3e), with total imports differing by **6.3%**^36^. These comparisons demonstrate that LNG-T3 reliably reproduces both temporal variability and regional trade dynamics, while also aligning closely with official annual aggregates.

### Uncertainty and bias

Although LNG-T3 shows strong agreement with reference data as shown above, several sources of uncertainty and bias are inherent in its construction. Our dataset relies heavily on AIS message processing, yet AIS reports are self-reported by vessels and may be subject to errors or delays. In addition, terrestrial AIS reception is only required in open-ocean regions, leading to gaps in trajectory coverage. Crucially, the reliability of terrestrial AIS is subject to the spatial density of receiver stations. Within the volunteer-based AISHub network, coverage is significantly more robust in the Global North (e.g., Europe and North America) than in regions like Southeast Asia, where sparser receiver nodes and archipelagic geography can lead to signal ‘blind spots.’ These issues introduce potential uncertainties, including (i) misclassification of vessel loading status due to inaccurate draught reporting, and (ii) missed or partially reconstructed voyages resulting from incomplete AIS tracks or missing port call records. While AIS and port call records are difficult to collect uniformly across all regions, this remains the most feasible and reliable approach for deriving global LNG trade flows from open-access sources. Moreover, we adopt a conservative strategy that retains only voyages validated for trade flow estimation. Together, these factors likely contribute to a systematic underestimation of total LNG trade volumes, which explains why LNG-T3 totals are generally smaller than industry and government statistics.

Another source of uncertainty arises from vessel cargo volume estimation. We assumed that all LNG tankers depart fully loaded, an approximation that facilitates global aggregation but does not reflect partial loadings. In practice, partial loadings may occur due to operational constraints, contractual arrangements, or market-driven adjustments. These cases could lead to overestimation at the individual-voyage level. Moreover, LNG-T3 captures physical transport flows only: it records where vessels load and unload LNG, without accounting for contractual ownership transfers or cargo redirections during transit. Such commercial transactions may introduce bias in country-to-country statistics, as our approach cannot track the contractual origin of the cargo. For example, a single mid-August LNG delivery cargo in 2024 was reported to have been traded up to 46 times in the spot market before final delivery^37^, highlighting how contractual chains can diverge from physical flows.

## Usage Notes

LNG-T3 provides a global inventory of LNG infrastructure (tankers and terminals) together with daily trade flows. The dataset supports analyses of infrastructure development, trade and market dynamics, and the role of key regional players. It improves transparency on LNG infrastructure and trade flows, enables quantitative studies of energy transitions and global supply chains, and supports assessments of GHG emissions from LNG transport under rapidly expanding demand. We provide the following examples to serve as a starting point on how the dataset can be used.

### Global LNG infrastructure expansion trends

The LNG tanker and terminal inventory from the LNG-T3 dataset enables detailed analysis of the evolution of LNG infrastructure. Fleet composition and terminal capacity define the physical limits of LNG trade, and their expansion trends are critical for assessing market flexibility and potential bottlenecks.

**Fig. 4:**
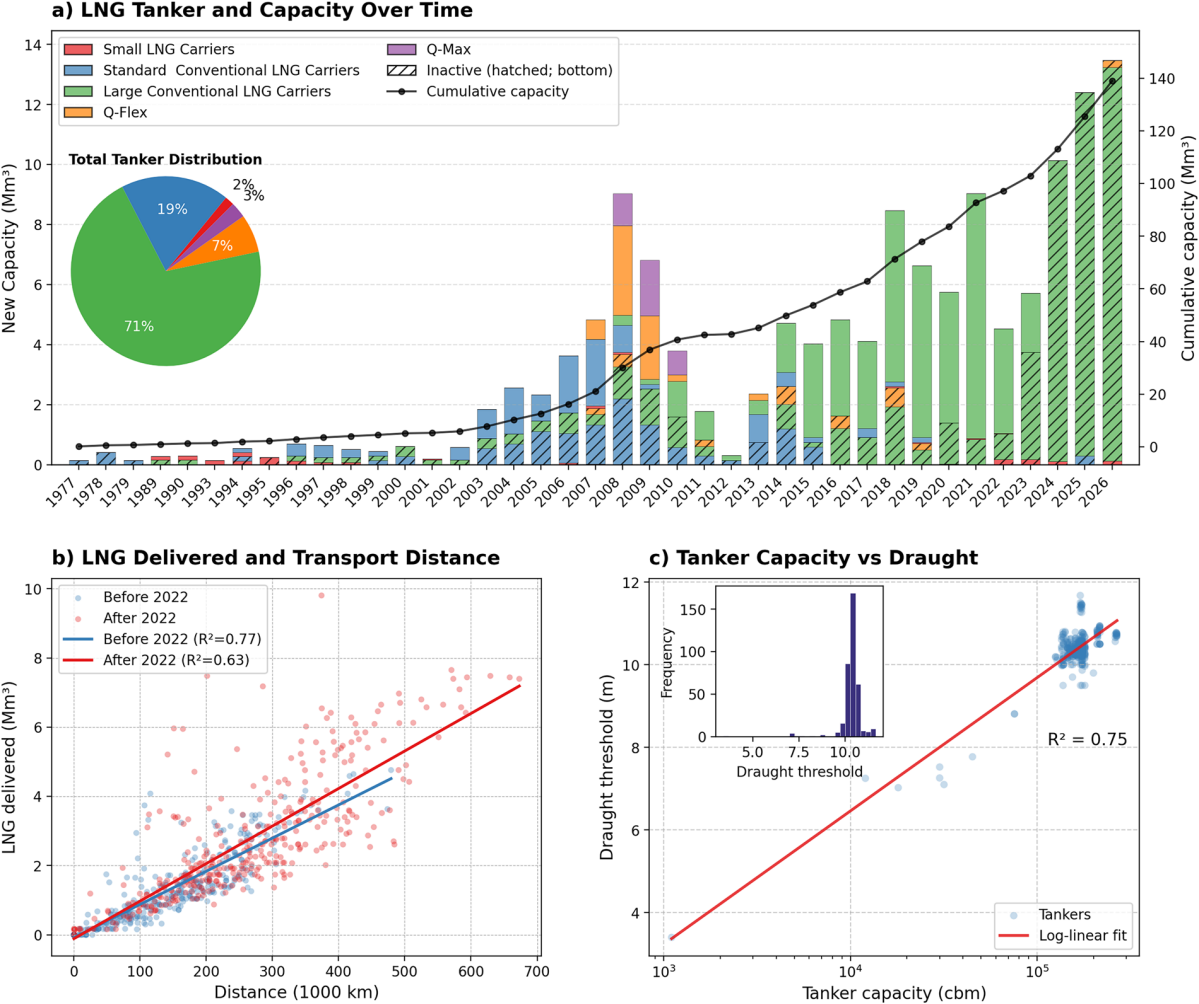
LNG Tanker Fleet Characteristics: (**a**) evolution of LNG tanker numbers and capacities over time, (**b**) relationship between LNG delivered and transport distance before and after 2022, (**c**) relationship between tanker capacity and draught thresholds, with inset histogram showing draught threshold distribution. The inactive tanker means those tankers has no AIS or port call data during the study periods (from 2020 to 2024). The tanker size category is defined in the **Method**.

Vessel records reveal two distinct surges in fleet expansion periods: the first between 2007 and 2010, dominated by Q-Flex and Q-Max vessels built to serve Qatar’s large-scale liquefaction projects, and the second between 2016 and 2022, when most new carriers were large conventional vessels rather than ultra-large ships (Fig. 4a). This second surge coincides with the rapid growth of U.S. LNG exports since 2016^38^, which required a more flexible fleet capable of serving diverse global routes. The trend away from ultra-large vessels toward versatile conventional carriers reflects the increasing geographical spread and heterogeneity of LNG trade in this period.

**Fig. 5:**
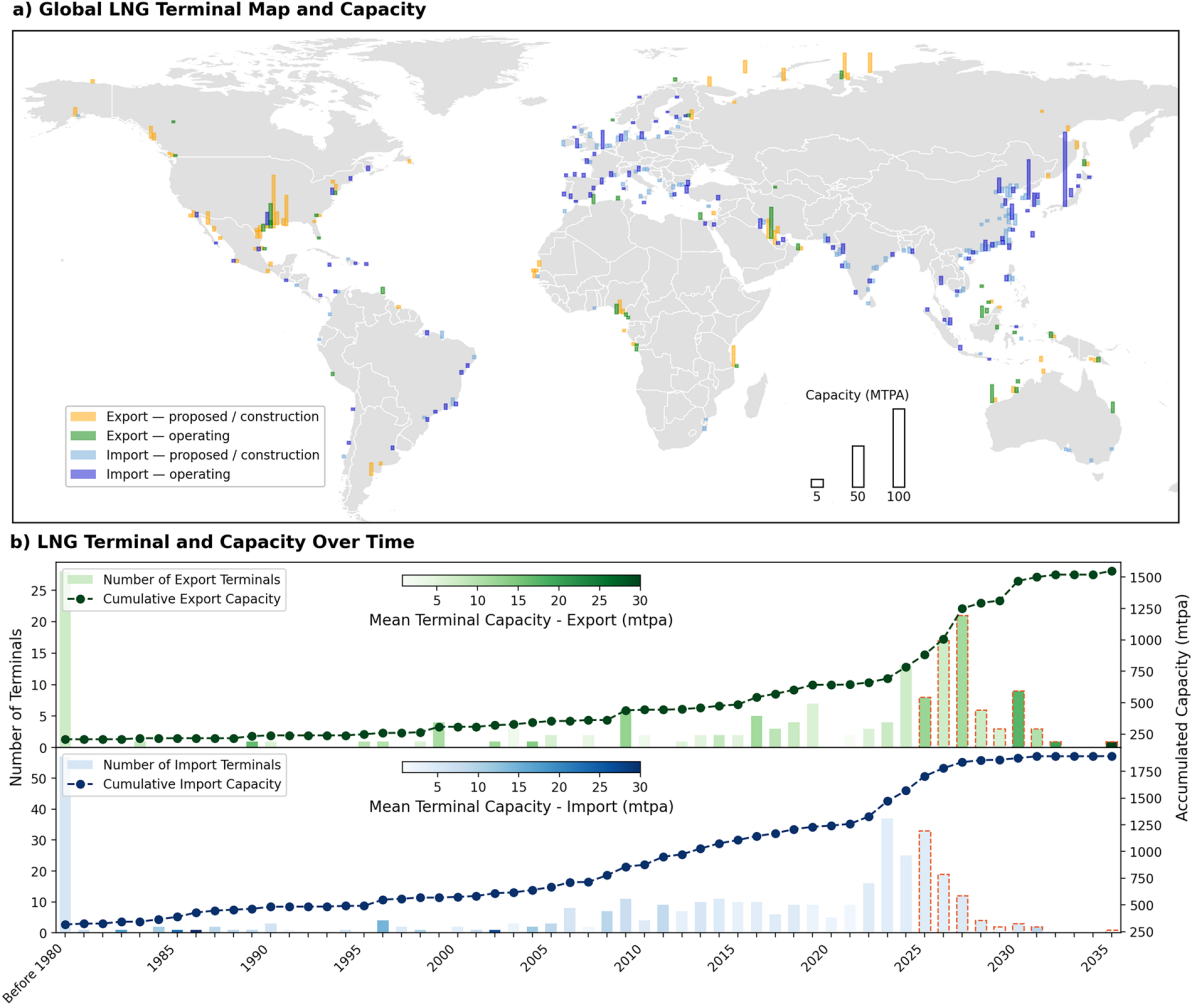
Global LNG Terminals: (**a**) distribution of LNG export and import terminals, including operating, under construction, and planned facilities, (**b**) number and cumulative capacity of export and import terminals over time. Terminal capacities are expressed in mtpa (million tonnes per year). To enhance the readability, the bars might represent multiple close terminals. The bars indicate the newly added number, while the scatter-line indicates the cumulative totals in plot (**b**).

On the terminal side, LNG-T3 shows steady global expansion of both export and import capacity before 2022, followed by a sharp surge thereafter, largely driven by Europe’s response to the Russian invasion of Ukraine and the loss of pipeline gas supplies (Fig. 5b). Since 2022, European countries have rapidly deployed new regasification capacity to diversify away from Russian gas. The largest share of new export terminals under construction is concentrated in the United States, reflecting its role as the primary replacement supplier to Europe (Fig. 5a), and in Russia, which is seeking alternative buyers for its natural gas.

### Maritime economics and climate impact

The integration of voyage distances and terminal-level throughput in LNG-T3 provides a robust foundation for interdisciplinary applications, particularly in maritime economics and climate policy. A comparison between pre- and post-2022 records reveal a significant shift toward longer routes characterized by larger per-delivery volumes, consistent with the Atlantic re-routing toward Europe following the 2022 energy crisis (Fig. 4b). Although the cumulative global sailing distance surged by **89.9%** due to higher overall trade volumes, the average transport intensity (km per m³ of LNG transported) actually decreased by **11.7%**. To statistically validate this shift, a Mann–Whitney U test was performed, which confirm a significant difference in transport intensity (amount per voyage distance) before and after 2022 (p < 0.01). These results indicate that larger cargo volumes per voyage partially offset the impact of longer routes at the aggregate level. However, a comprehensive evaluation of the overall climate implications of expanding LNG trade would require combining transport emissions, terminal methane leakage, and comparisons with alternative gas delivery pathways, which is supported by the high-resolution activity data provided in LNG-T3.

**Fig. 6:**
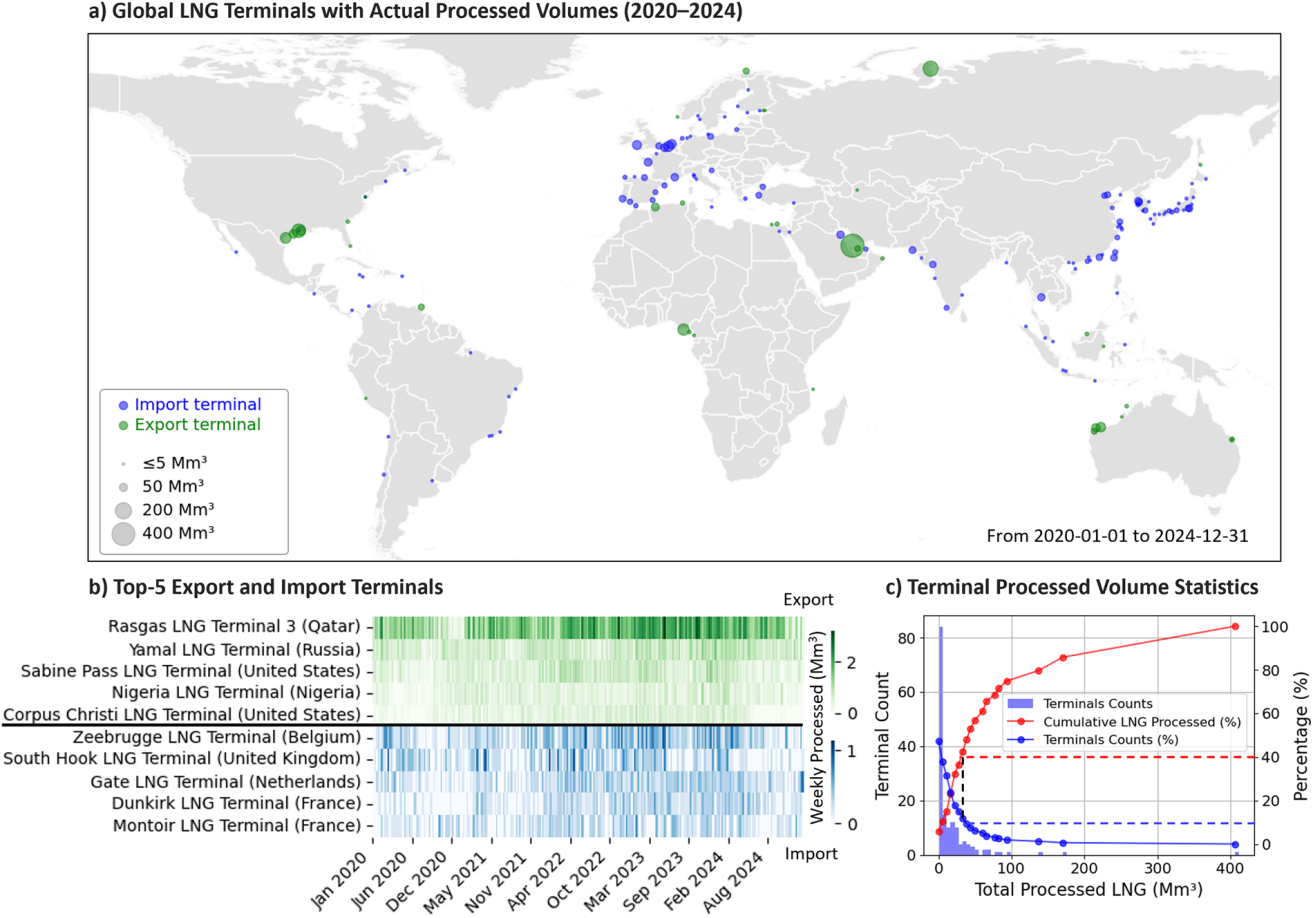
Global LNG Terminal Activity and Processed Volumes (2020–2024): (**a**) distribution of export and import terminals with actual processed LNG volumes, (**b**) weekly processed volumes at the top five export and import terminals, (**c**) distribution of terminal processed volumes, showing terminal counts and cumulative shares.

Furthermore, LNG-T3 highlights that global terminal usage remains highly concentrated (Fig. 6b,c), with the top ten export and import terminals—including major hubs such as Rasgas (Qatar), Yamal (Russia), Sabine Pass (U.S.), Zeebrugge (Belgium), and South Hook (UK)—accounting for approximately **40%** of global throughput. These high-resolution activity records can be integrated with satellite-based methane observations (e.g., TROPOMI) to investigate potential correlations between terminal utilization intensities and localized atmospheric CH_4_ emissions. Such cross-disciplinary utility demonstrates that LNG-T3 is a critical tool for quantifying the intersection of global trade logistics and infrastructure-level environmental monitoring.

### Tracking global market dynamics and shock responses

**Fig. 7:**
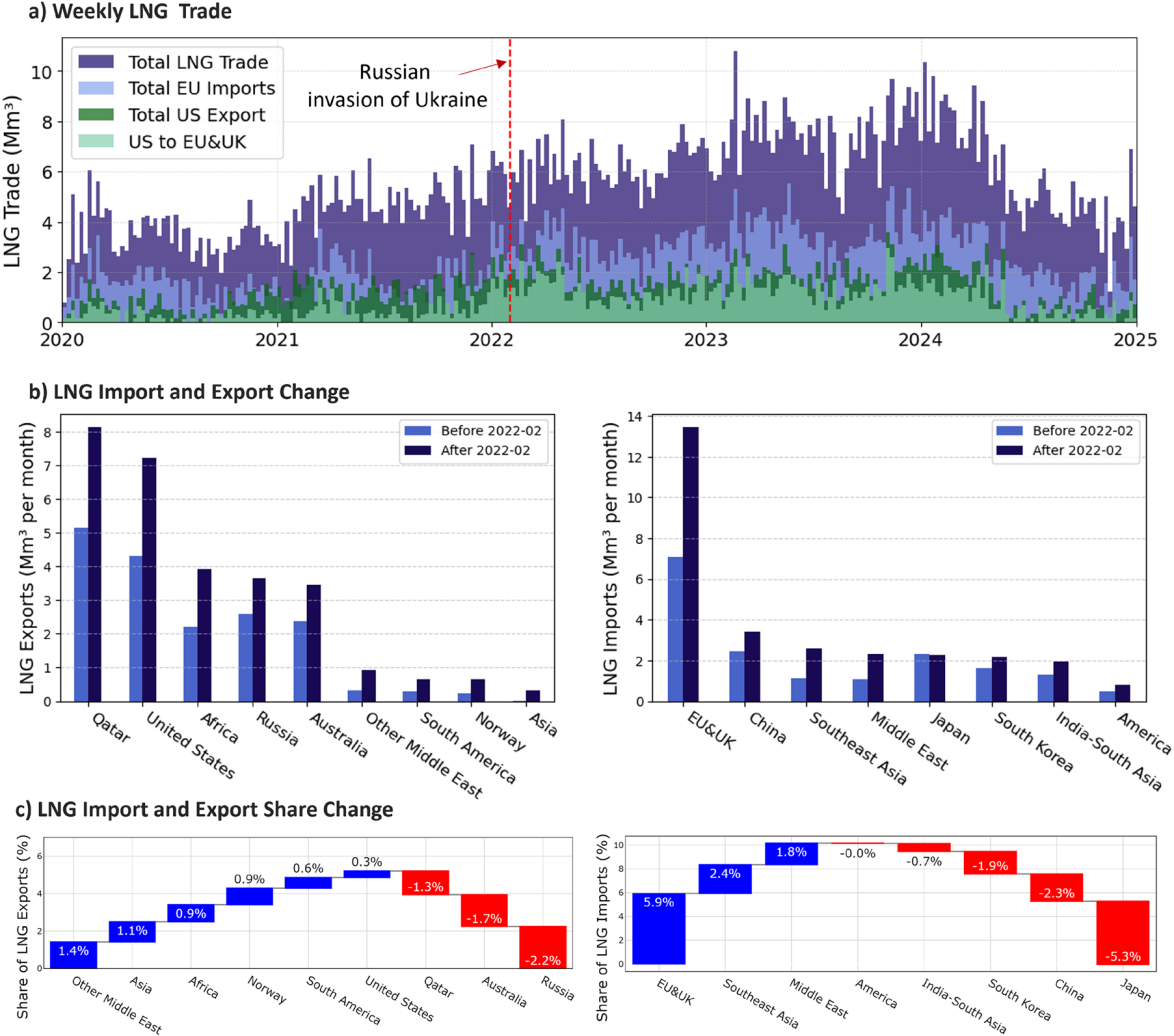
Global LNG Trade Dynamics and Regional Shifts: (**a**) weekly LNG trade volumes, (**b**) monthly LNG export and import volumes before and after February 2022, (**c**) changes in regional shares of global LNG exports and imports before and after February 2022.

The LNG-T3 dataset reveals a steadily expanding LNG market, with an overall increase of 58.6% in 2024 compared to 2020 (Fig. 7a). By providing daily country-to-country flows, the dataset enables analysis across multiple time scales and reveals temporal dynamics often masked in monthly or annual statistics. For example, weekly global LNG trade rose sharply following the Russian invasion of Ukraine in February 2022, with EU&UK imports showing the largest and most sustained increase. While most major exporters expanded shipments (Fig. 7b), the EU&UK were the only importers with a significant surge, positioning Europe as the central driver of global market adjustment (Fig. 7b–c). Detailed country-to-country flows also reveal the emergence of new exporters to the EU after the invasion, such as Cameroon, Turkmenistan, and Peru (Table 5), which indicates that Europe sought supplier diversity and that smaller producers began to enter the global LNG market. While these observed shifts align with the timing of the conflict, it should be noted that this descriptive analysis does not formally decouple specific geopolitical shocks from broader, underlying trends in the global energy transition. These shifts underscore the invasion’s pivotal role in reshaping LNG trade flows, strengthening the Atlantic basin, and accelerating the ongoing transformation of the global LNG market.

**Table 5:** Change of LNG supplier to EU&UK before and after the Russia invasion of Ukraine (2022-02).

EU&UK Share of Global Imports (%)	Pre-Invasion		Post-invasion	
	40.5		46.4	
Supplier	Share of EU&UK Imports (%)	EU&UK Share of Supplier Exports (%)	Share of EU&UK Imports (%)	EU&UK Share of Supplier Exports (%)
United States	24.2	39.7	**36.7**	**68.1**
Russia	**30.8**	**84.1**	22.8	**84.3**
Qatar	23.3	32.1	15.5	25.7
Nigeria	10.4	**56.2**	8.4	**62.1**
Algeria	6.3	**63.5**	5.8	**61.2**
Norway	3.2	**94.9**	4.6	**95.7**
Egypt	0.4	39.8	2.0	**56.0**
Trinidad and Tobago	1.0	25.1	1.7	38.0
Equatorial Guinea	0.5	36.8	0.5	30.6
Cameroon	\	\	0.7	**63.4**
Turkmenistan	\	\	0.6	**54.6**
Peru	\	\	0.3	**53.5**
Other New Suppliers*	\	\	0.5	1.5

*Other new suppliers include Australia, the United Arab Emirates, Malaysia, and Oman.

### Regional key players and shifting trade flows

**Fig. 8:**
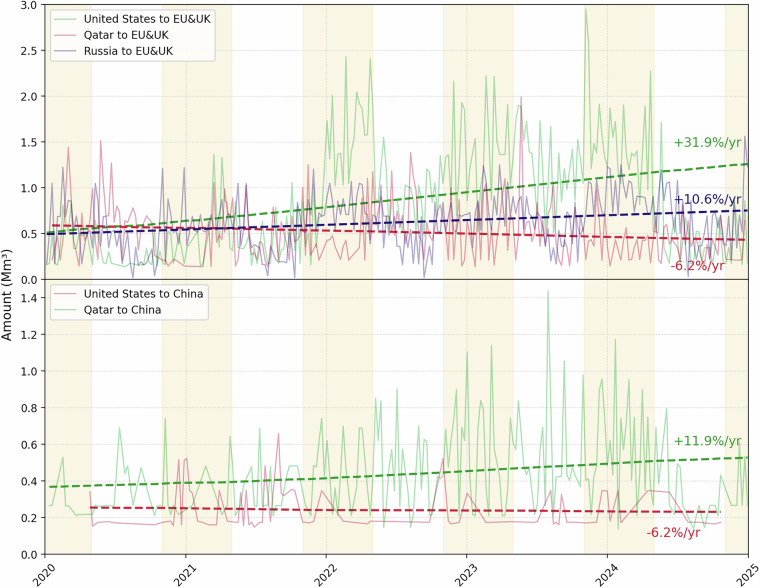
LNG Trade Flows and Growth Trends (2020–2024) for LNG exports from the United States, Qatar, and Russia to the EU&UK (top), and from the United States and Qatar to China (bottom).

The LNG-T3 dataset enables route-specific tracking of LNG flows, revealing how supplier–consumer relationships evolve over time. Such insights into emerging dependencies and diversification are critical for understanding energy security and the geopolitical impact. Since 2020, U.S. exports to EU&UK have grown most rapidly (+31.9%/yr, Fig. 8 top), underscoring the region’s strategic pivot to American LNG. Russian exports to EU&UK, despite the invasion of Ukraine, have continued to increase (+10.6%/yr, Fig. 8 top), highlighting that Russia remained a primary source of gas supply to Europe despite pipeline closures and sanctions. The EU’s continued import of LNG from Russia might reflect the limited availability of alternative global suppliers and the lasting impacts of the energy crisis. By contrast, Qatar’s exports to EU&UK have declined (−6.2%/yr, Fig. 8 top), with a redirection of Qatari volumes toward Asia. China has absorbed more LNG from Qatar (+11.9%/yr, Fig. 8**bottom**) while reducing imports from the U.S. (−6.2%/yr, Fig. 8**bottom**). While this use case example highlights major supplier-to-consumer shifts, it should be noted that a comprehensive decomposition of internal regional import shares that would provide granular insights into localized energy dependencies is also supported by this dataset.

The observed reallocation patterns highlight the relative flexibility of seaborne LNG compared to pipeline-based gas trade, enabling rapid adjustment to regional supply-demand imbalances. The LNG-T3 dataset serves as an essential foundation for further evaluating the role of LNG as a potential “bridge fuel” in the decarbonization pathway, particularly regarding its capacity to mitigate energy supply shocks through its unique geographical flexibility.

## Data Availability

The LNG-T3 dataset is openly available through **Zenodo** at 10.5281/zenodo.17273526^39^. The repository contains the full dataset in CSV format, including the LNG tanker inventory, LNG terminal inventory, daily terminal-level activity, daily country-to-country trade flows, and the supplementary reference data from GIIGNL reports. Metadata descriptions and variable definitions are provided both in Tables 2, 3 of this paper and in the Zenodo repository page. The current release (v1.0) covers January 2020 to December 2024 and is archived as a fixed version on Zenodo. Future updates, if implemented, will be released as separate versioned datasets with extended temporal coverage. The underlying data structure and variable definitions will remain consistent to ensure comparability across versions.

## Code availability

Source code for AIS data collection and visualization is provided alongside the dataset. Scripts related to the processing of commercial port call data are available from the corresponding author upon reasonable request.
